# Decoding acute myocarditis in patients with COVID-19: Early detection through machine learning and hematological indices

**DOI:** 10.1016/j.isci.2023.108524

**Published:** 2023-11-23

**Authors:** Haiyang Li, Zhangkai J. Cheng, Xing Fu, Mingtao Liu, Peng Liu, Wenhan Cao, Zhiman Liang, Fei Wang, Baoqing Sun

**Affiliations:** 1 Department of Clinical Laboratory, National Clinical Research Center of Respiratory Disease, Guangzhou Institute of Respiratory Health, First Affiliated Hospital of Guangzhou Medical University, Guangzhou Medical University, Guangzhou 510120, China; 2 Department of Clinical Pharmacy, Dazhou Central Hospital, Dazhou 635000, China; 3 Group of Theoretical Biology, School of Life Sciences, Sun Yat-sen University, Guangzhou 510275, China; 4 MRC Biostatistics Unit, University of Cambridge, Cambridge CB2 0SR, UK

**Keywords:** Health sciences, Diagnostics, Artificial intelligence

## Abstract

During the persistent COVID-19 pandemic, the swift progression of acute myocarditis has emerged as a profound concern due to its augmented mortality, underscoring the urgency of prompt diagnosis. This study analyzed blood samples from 5,230 COVID-19 individuals, identifying key blood and myocardial markers that illuminate the relationship between COVID-19 severity and myocarditis. A predictive model, applying Bayesian and random forest methodologies, was constructed for myocarditis' early identification, unveiling a balanced gender distribution in myocarditis cases contrary to a male predominance in COVID-19 occurrences. Particularly, older men exhibited heightened vulnerability to severe COVID-19 strains. The analysis revealed myocarditis was notably prevalent in younger demographics, and two subvariants COVID-19 progression paths were identified, characterized by symptom intensity and specific blood indicators. The enhanced myocardial marker model displayed remarkable diagnostic accuracy, advocating its valuable application in future myocarditis detection and treatment strategies amidst the COVID-19 crisis.

## Highlights

• Younger people face higher myocarditis risk
• Omicron EG.5 variant has higher myocarditis risk than BA.5.2
• Multi-index model (AUC 0.924) improves early myocarditis detection
• COVID-19 shows unique markers; low ALB/GLB in severe cases helps stratify

## Graphical abstract

**Figure:**
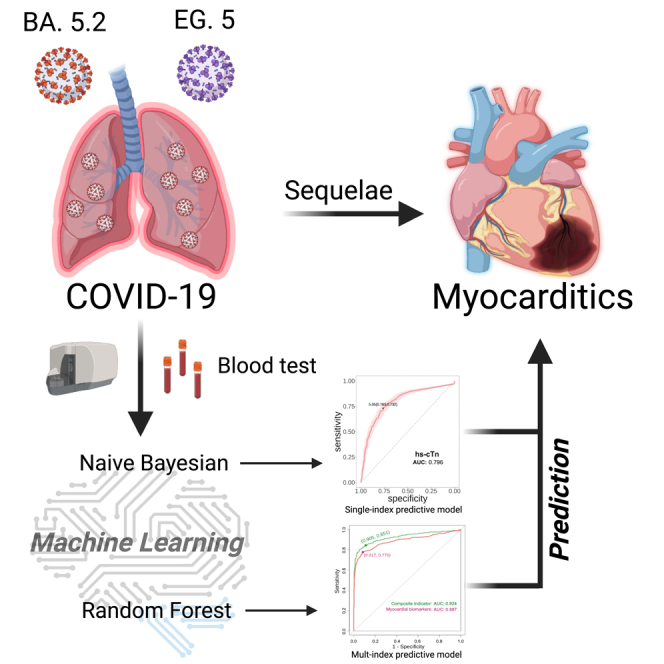

## Introduction

The coronavirus disease 2019 (COVID-19) has swept almost the world’s population. It is a pandemic infectious disease caused by the severe acute respiratory syndrome coronavirus 2 (SARS-COV-2).^1^^,^^2^ As the disease progresses, it can lead to a range of symptoms and complications.^3^ It will also produce different degrees of sequela after the patient recovers.^4^^,^^5^ Among them, there is now increasing evidence that acute viral myocarditis is a highly prevalent and fatal sequela of COVID-19,^6^ especially in young adult and adolescent males. In the coming years, we may also be affected by COVID-19 in the long term. Therefore, predicting myocarditis in patients is particularly important for the health of people and even adolescents.

Acute myocarditis is a recognized complication of acute viral infection^7^ which can have serious consequences, including heart failure, arrhythmias, dilated cardiomyopathy, sudden cardiac death, and long-term complications. Relevant studies on prognostic indicators of myocarditis include cardiac troponin-I and homocysteine.^8^ Prompt diagnosis and treatment are important to minimize the harm caused by this condition. Hendrickson et al.^9^ conducted a follow-up investigation on the athletes infected with COVID-19 and found that nearly 50% of the athletes had cardiac function defects in the prognosis. However, other studies have suggested that the incidence of myocarditis may be higher in certain subgroups of patients. Modin et al.^10^ points out that 15% had evidence of myocardial injury, which can sometimes lead to myocarditis. It is important to note that the available data on myocarditis in patients with COVID-19 is still evolving, and more research is needed to understand the risks and outcomes associated with this complication fully. Additionally, factors such as age, sex, and underlying medical conditions may play a role in the incidence of myocarditis in patients with COVID-19.^11^

Significantly, acute myocarditis and COVID-19 have been shown to affect the blood routine index, including white blood cell count, red blood cell count, and platelet count. Y. Liu, Y. Yang et al.^12^ found that patients with COVID-19 have lower lymphocyte and platelet levels than healthy controls. The findings suggest that these blood cell count abnormalities may be associated with the severity of the disease and could potentially serve as prognostic indicators. Terpos et al.^13^ analyzed the hematological parameters of patients with COVID-19 and found that they had higher levels of inflammatory markers, such as C-reactive protein (CRP) and erythrocyte sedimentation rate (ESR), as well as lower levels of hemoglobin and red blood cells compared to healthy controls. They also observed that patients with severe COVID-19 had higher CRP and ESR levels and lower hemoglobin and red blood cell counts than those with mild cases. The findings suggest that these hematological abnormalities may be associated with the severity of COVID-19 infection and could potentially serve as prognostic indicators. These changes in blood parameters may be related to the inflammatory response to the virus and can help to identify patients with severe disease.^14^ At present, much work has been devoted to finding relevant markers and patient indicators to study COVID-19 and predict the Sequelae of COVID-19.^15^ Ullah et al.^16^ studied the predictive correlation between the lymphocyte-to-neutrophil ratio (LNR) in patients with COVID-19 and found that high LNR had a higher probability of predicting in-hospital mortality. Tamaki et al.^17^ investigated the indicators of 1026 patients with heart failure and conducted follow-ups. These results verify the combined role of LNR and lymphocyte-to-platelet ratio (LPR) in evaluating the prognosis of patients with acute heart failure. Along with albumin^18^ peripheral lymphocyte count^19^^,^^20^^,^^21^ lymphocyte to neutrophil ratio^22^ their research proved changes in blood routine indicators are commonly observed in patients with COVID-19. It may provide important diagnostic and prognostic information. However, it is essential to note that these changes may vary among patients and should be interpreted in the context of the patient’s overall clinical picture. Further research is needed to understand the mechanisms underlying these changes fully and to develop more effective treatments for COVID-19.

**Figure 1:**
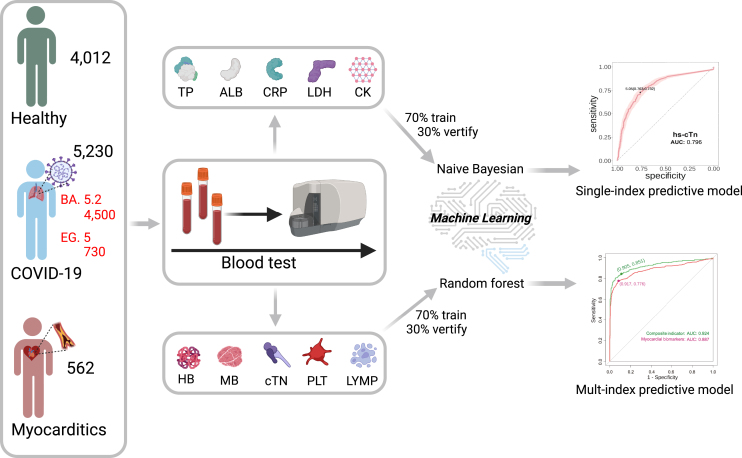
The scheme of analysis and prediction of indexes of COVID-19 and myocarditis The blood samples were collected from a diverse group of individuals, including 5,230 patients diagnosed with COVID-19 (4500 BA.5.2 & 730 EG.5), 562 individuals suffering from myocarditis, and approximately 4,012 healthy individuals. These samples were then analyzed to identify various biochemical indicators and biomarkers. The data derived from this analysis was subsequently input into a statistical model. This model was designed to systematically predict both the diagnosis and prognosis of myocarditis, providing a potentially powerful tool in the fight against this condition.

Here, we collected blood samples from 5,230 patients with COVID-19, 4,012 healthy individuals, and 562 patients with myocarditis recovered from COVID-19. We measured patients’ blood indicators, biochemical indicators and related markers and then further compared the differences between patients with COVID-19 and myocarditis. We trained the classification predictive model according to the relevant data to explore how patient indicators change to develop myocarditis. Figure 1 shows the experimental and control groups’ routine biochemical and specific indicators. Then, a naive Bayesian model and random forest (RF) machine learning algorithm were applied to the training of index data of sample blood, to construct receiver operating characteristic (ROC) spectrum of subjects, and to calculate the area under the curve (AUC) to distinguish the condition of regular and patients with myocarditis. Further correlation analysis was conducted between COVID-19 and the indicators of patients with myocarditis to predict the patients with COVID-19 at risk of myocarditis, thereby improving the survival rate of patients. At the same time, we performed a thorough analysis of the correlation and difference of indicators, as well as the distribution of ratios of severe indicators.

## Results

### The demographic characteristics of all samples

**Figure 2:**
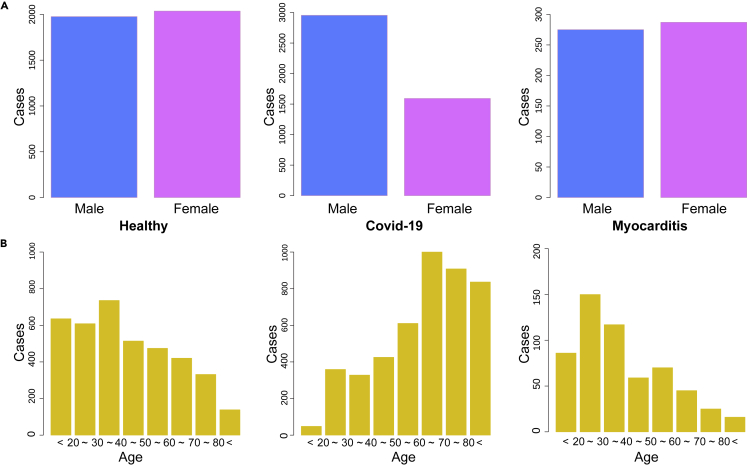
Gender and age distribution of the study sample (A) The sexual distribution of the healthy population, patients with COVID-19 (BA.5.2), and myocarditis. It displayed the number of men and women in each participating population. (B) The age distribution of healthy population, patients with COVID-19 (BA.5.2), and myocarditis. Statistics the number of people in each age group.

In this study, the gender ratio of males to females in both the patients with myocarditis and the healthy population is approximately balanced at 1:1. The average age of the healthy population is 38 years, with a standard deviation of 20 years. The age range for patients with myocarditis is 39 ± 22 years. Both groups share the same median age of 35 years within the IQR. In contrast, among patients with COVID-19, the male-to-female ratio is 2810:1690 (Figure 2A). The average age for this group is higher, at 65 years, with a standard deviation of 20 years. Moreover, the median age within the IQR is also higher, at 70 years. Females account for 37.6% of patients with COVID-19, a proportion notably lower than that of males. This contrasts with the myocarditis and healthy groups, where the gender ratio is essentially balanced. Table 1 presents the detailed demographic characteristic information. It is worth noting that the age distribution among the healthy population is more uniform across age ranges. In contrast, the age distribution among patients with COVID-19 is more concentrated in the older age group (60–80 years). Conversely, patients with myocarditis are primarily concentrated in the younger age group (20–40 years), as shown in Figure 2B.

**Table 1:** The sample information and blood indicator of healthy population, patients with COVID-19, and myocarditis

	Healthy	COVID-19 (BA.5.2)	Myocarditis	p value (COVID-19 VS Myoca.)
Male	1976 (49.3%)	2810 (62.4%)	275 (48.9%)	
Female	2036 (50.7%)	1690 (37.6%)	275 (51.1%)	
Age	35 (27,49)	70 (56,81)	35 (22,56)	<0.001
TP	76 (72,79)	65 (59,74)	65 (60,71)	0.5
ALB	44 (43,46)	36 (32,42)	37 (32,40)	0.7
hs-CRP	1 (1,2)	19 (5,53)	5 (1,27)	<0.001
CRP	0.3 (0.1,0.7)	0.3 (0.1,1.8)	2.3 (0.6,8.1)	<0.001
LDH	166 (145,190)	256 (200,360)	217 (168,329)	<0.001
CK	76 (78,145)	73 (41,130)	90 (55,161)	<0.001
HB	142 (129,153)	113 (91,131)	120 (99,133)	<0.001
MB	19 (15,22)	36 (20,92)	33 (8,51)	<0.001
hs-cTn	2 (2,3)	8 (3,21)	27 (3,78)	<0.001
PLT	241 (206,291)	218 (154,286)	230 (165,298)	0.008
LYMP%	33 (28,39)	13 (6,25)	19 (9,29)	<0.001

Interquartile range and t-test, where a statistically significant difference is p value shown in table.

The levels of various blood indicators exhibited significant variations between groups, as detailed in Table 1, showing the main indicators are the results of the quartile data, presented in the form of the median (IQR: 25%, 75%). The p value represents the Kruskal-Wallis test between patients with COVID-19 and myocarditis. The comprehensive indicators for each group demonstrate extreme statistical significance, with only TP and ALB not showing statistical significance. Furthermore, each index’s corresponding values display the indicators’ average value and fluctuation range (interquartile range) in different groups. Among all indicators, the indicator level of healthy people is significantly different from that of patients with COVID-19 and myocarditis. However, it can be challenging to fully comprehend the relationships between data through numerical values in Table 1 alone. Therefore, in the following analysis, we plan to conduct a more in-depth analysis of various indicators using different statistical methods.

### Statistical analysis of blood protein indicators

As a prospective biomarker, the albumin/globulin (ALB/GLB) ratio (AGR) could be instrumental in risk categorization and management of patients with COVID-19. Prompt identification of patients exhibiting a lower AGR could enable early potentially mitigating or managing severe disease progression. Additionally, tracking the AGR throughout the disease’s duration could offer valuable insights into the patient’s response to treatment and the path of recovery. The AGR essentially mirrors the balance between the body’s inflammatory and anti-inflammatory responses. A diminished AGR signifies an increased the production of globulins, specifically immunoglobulins and acute-phase proteins, in reaction to infection and inflammation. This amplified immune response can trigger severe inflammation and tissue damage in patients with COVID-19, leading to poorer clinical outcomes. Furthermore, ALB, a negative acute-phase protein, decreases its concentration during infection and inflammation. A decreased ALB level could be indicative of malnutrition, which can exacerbate outcomes in patients with COVID-19. Therefore, the AGR can serve as an indicator of both nutritional status and disease severity.

**Figure 3:**
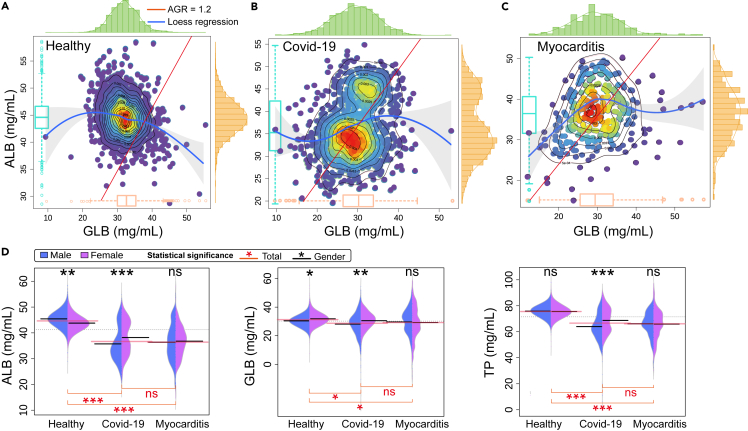
The distribution of ALB and GLB with statistically significant analysis (A) The distribution of ALB/GLB ratios in a healthy population. The red line represents the threshold with an AGR of 1.2. The boxplot indicates the mean ± SD, and the blue line depicts the loess regression across all data points, with the surrounding shaded area representing the confidence interval. (B) The ALB/GLB distribution of COVID-19. The red line is the dividing line of AGR = 1.2. The boxplot indicates the mean ± SD, and the blue line is the loess regression at all points, the shadow is the confidence interval. (C) The ALB/GLB distribution of patients with myocarditis. The red line is the dividing line of AGR = 1.2. The boxplot indicates the mean ± SD, and the blue line is the loess regression at all points, the shadow is the confidence interval. (D) The ALB, GLB, TP distribution and statistical significance of healthy population, patients with COVID-19, and myocarditis. Interquartile range and t-test, where a statistically significant difference is denoted by ∗∗∗ (p < 0.001), ∗∗(p < 0.01), and ∗ (p < 0.05), and non-significance (p > 0.05) is denoted by "ns".

The AGR with a cutoff value of 1.2 has been identified as a highly sensitive combination marker.^23^ This is visually represented in Figure 3A–3C, where the red line denotes an AGR of 1.2. The blue line, fitted using loess regression, provides a fundamental representation of the two-dimensional data distribution. Notably, the scatter points for the healthy population predominantly lie above the AGR = 1.2 line, while a more significant proportion of scatter points for patients with COVID-19 and myocarditis are found below this line, this suggests a clear differentiation in the AGR between healthy individuals and those with COVID-19 or myocarditis, which could serve as a valuable tool for early detection and intervention. These scatter points are presented as a heatmap, where the red area indicates concentrated points of the indicator and the purple area represents scattered points. The proportion of asymptomatic youth was higher, and the results showed higher TP indicators (Figure S1). This visual representation provides an intuitive way to understand the distribution of these indicators, which could be helpful in both clinical and research settings. The peripheral bar chart complements this by visually representing the concentration of scatter points in the relative area. This allows for a straightforward comparison of the density of data points across different ranges of AGR. Interestingly, the hotspot center, which represents the area with the highest concentration of data points, aligns with the peak distribution of the bar chart and the boxplot located in the red area. This consistency across different visual representations reinforces the reliability of these findings.

Upon analyzing the results depicted in Figure 3D, it was observed that the ALB levels in patients with COVID-19 and myocarditis were notably lower compared to those in the healthy population. Interestingly, the ALB levels in patients with COVID-19 and myocarditis were virtually identical, with no statistical significance between them. Concurrently, it was noted that the trends in TP and ALB levels were in line with these findings. The globulin level appeared to be unaffected by the disease, with all three groups maintaining a consistent level. Subsequently, upon comparing indicators between male and female samples, we found no such statistically significant was observed in ALB, GLB, and TP indicators among patients with myocarditis of both genders. Similarly, TP levels showed no significant variation between healthy men and women. Notably, the gender-based difference in protein levels was most pronounced in patients with COVID-19.

### The risk factors in COVID-19 and myocarditis

**Figure 4:**
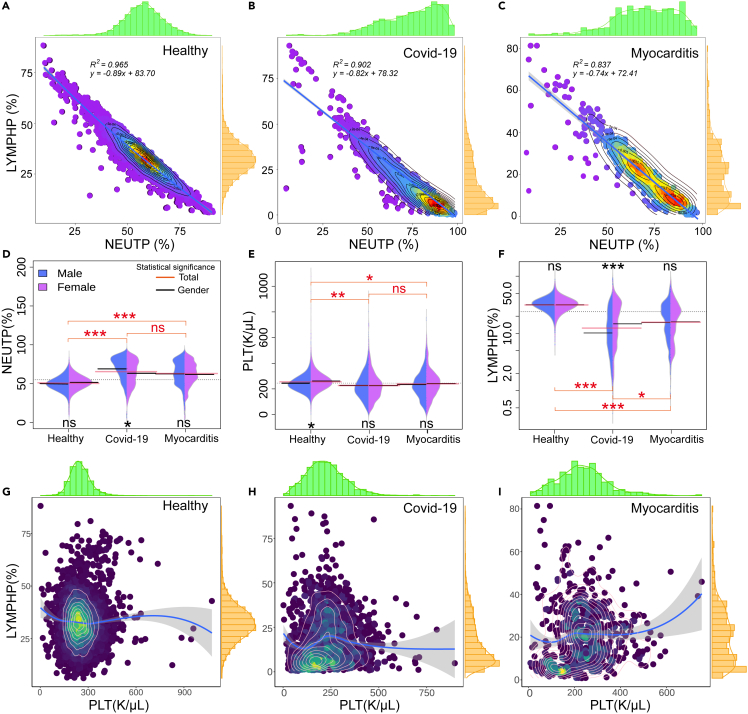
The blood index distribution of LNR, LPR, and individual indicators (A) The blood index distribution of LNR in the healthy population. (B) The blood index distribution of LNR in the COVID-19. (C) The blood index distribution of LNR in the patients with myocarditis. Linear regression. (D) The distribution and Statistical significance analysis of the healthy population. (E) The distribution and Statistical significance analysis of the COVID-19. (F) The distribution and Statistical significance analysis of the patients with myocarditis. (G) The blood index distribution of LPR in the healthy population. (H) The blood index distribution of LPR in the COVID-19. (I) The blood index distribution of LPR in the patients with myocarditis. The blue line is the loess regression at all points, the shadow is the confidence interval. Interquartile range and t-test, where a statistically significant difference is denoted by ∗∗∗ (p < 0.001), ∗∗(p < 0.01), and ∗ (p < 0.05), and non-significance (p > 0.05) is denoted by "ns".

The lymphocyte-to-neutrophil ratio (LNR) has been identified as an independent risk factor for mortality in hospitalized patients with COVID-19.^24^ This ratio, calculated from routine blood tests, measures systemic inflammation and immune response. Several studies have found that patients with severe COVID-19 often have a lower LNR compared to those with mild disease. A lower LNR may have been associated with more severe disease. In some patients with severe COVID-19, there can be a change in the ratio of lymphocytes to neutrophils, often with an increase in neutrophils and a decrease in lymphocytes. This is often referred to as the lymphocyte-to-neutrophil ratio (LNR). This is likely due to the increased neutrophil count, a response to infection and inflammation, and decreased lymphocyte count, which could result from viral-induced lymphocyte apoptosis or redistribution. In the scatter analysis of neutrophils and lymphocytes, the sample distribution showed a good linear relationship and a linear model fitted the relevant data. The results were shown in Figures 4A–4C, and the healthy population showed a good fit. The R2 of patients with COVID-19 and myocarditis decreased successively, which was mainly due to the disease-causing the spread of scatter distribution. After linear fitting, we obtained the slope of the fitting equation, with health being −0.83, COVID-19 being −0.82, and myocarditis being −0.74. The slope of the corresponding law is an increasing relationship. L/N shows a rising disease trend and is more prevalent in patients with more severe myocarditis. Through the scattered density heat analysis, it can be found that the healthy people are mainly concentrated near the midpoint of the ratio. In contrast, the density hot spots of COVID-19 and myocarditis are concentrated in the low lymphocyte area, myocarditis has two density concentration areas, and the bar chart also shows two peaks.

The results of single indicator analysis showed indicators such as NEUTP (Figure 4D), PLT (Figure 4E), and LYMPHP (Figure 4F) between men and women. Only the LYMPHP of patients with COVID-19 showed statistical significance, while the results of other indicators did not show statistical significance. In the three groups, the three indicators of patients with COVID-19 and myocarditis basically did not have statistical significance. In comparison, the healthy people and these two groups had extremely high statistical significance. Early studies suggested that the LPR could be a valuable tool in predicting the severity of COVID-19. Patients with severe forms of the disease appeared to have a lower LPR compared to those with milder forms.^25^ These ratios, often referred to as the LPR, can serve as potential biomarkers for disease prognosis and treatment response. Figures 4G–4I, In the context of COVID-19, (Figure 4H), a decrease in lymphocyte count (lymphopenia) and an increase in platelet count (thrombolysis) have been frequently observed. This imbalance, reflected in a low LPR, could indicate a severe inflammatory response and hypercoagulable state. However, while the LNR is a promising tool, it is not without limitations. It can be influenced by various factors, including age, sex, and comorbidities, and it is not specific to COVID-19. Therefore, it should be used in conjunction with other clinical parameters and biomarkers to assess the risk of severe disease and mortality in patients with COVID-19.

Our analysis highlights the concurrence between the number of lymphocytes and the LYMPHP distribution (Figure S2A). Moreover, the hemoglobin (HB) index in healthy individuals is markedly higher than that observed in patients suffering from COVID-19 and myocarditis. This disparity offers crucial insight into the pathophysiology of these conditions and their impact on normal body functioning. Equally significant is the elevated inflammatory marker C-reactive protein (CRP) observed in patients with COVID-19, surpassing levels detected in patients with myocarditis. This considerable change indicates the body’s intense inflammatory response in response to COVID-19 infection. Such an inflammatory surge could potentially exacerbate disease severity, which warrants further investigation. The statistical significance analysis (Figure S2B) indicates that barring CRP levels in patients with COVID-19 and myocarditis, all other examined indicators exhibited statistically significant differences between the groups. This distinction in indicators could serve as a pivotal tool in distinguishing between COVID-19 and myocarditis, assisting in timely and accurate diagnosis and, thus, appropriate treatment.

Furthermore, correlation analyses among indicators in different groups (Figure S3) reveal a higher incidence of negative correlation amongst indicators in patients with COVID-19 and myocarditis, which could suggest a complex interplay of factors in these diseases. This relationship necessitates a more comprehensive exploration. Moreover, the correlation scatter analysis (Figure S5) shows that the indicators of patients with myocarditis largely overlap with the range of patients with COVID-19. This overlap might underscore the standard pathophysiological processes in both conditions and may have implications for shared therapeutic approaches. These findings illuminate significant patterns and correlations among critical indicators in patients with COVID-19 and myocarditis. They hold the potential to facilitate a better understanding of these diseases, aid in the development of diagnostic tools, and inform effective therapeutic strategies.

### Statistical analysis of myocardial indicators

**Figure 5:**
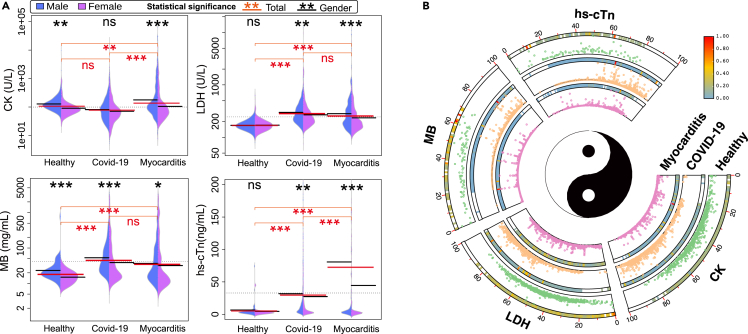
The distribution and statistical significance analysis results of cardiac markers (CK, LDH, MB, hs-cTn) in the healthy population, COVID-19, and myocarditis patient’s groups (A) Gender statistical significance distribution. Interquartile range and t-test, where a statistically significant difference is denoted by ∗∗∗ (p < 0.001), ∗∗ (p < 0.01), and ∗ (p < 0.05), and non-significance (p > 0.05) is denoted by "ns". (B) The age distribution of relevant myocardial indicators in each group (normalized).

Research into myocardial biomarkers such as CK, LDH, MB, and hs-cTn has underscored their potential importance in predicting myocarditis. The distribution of myocardial-related indicators (Figure 5). We compared the myocardial markers of healthy individuals, patients with COVID-19, and patients with myocarditis. The results showed the distribution of indicators in distinct populations and differences in indicators between male and female samples. It was found that the levels of CK, LDH, MB, and hs-cTn (Figure 5A) in patients with myocarditis were significantly elevated compared to the healthy population, all displaying a highly significant statistical difference. Moreover, for patients with COVID-19 and myocarditis, the LDH and MB indicators were essentially consistent, demonstrating no statistical significance. We also visualized the distribution of age and myocardial-related markers at scattered points for each group using Figure 5B. The heat maps and scatterplots were presented for each group. The narrowed heat maps represent datasets from different age groups. After normalization, the scattered points allow for the comparison of healthy individuals, patients with COVID-19, and patients with myocarditis. This comprehensive analysis of multiple biomarkers, along with the visualization of their distribution across different age groups and populations, provides valuable insights for diagnosing and managing myocarditis.

### Performance of a predictive model for myocarditis

**Figure 6:**
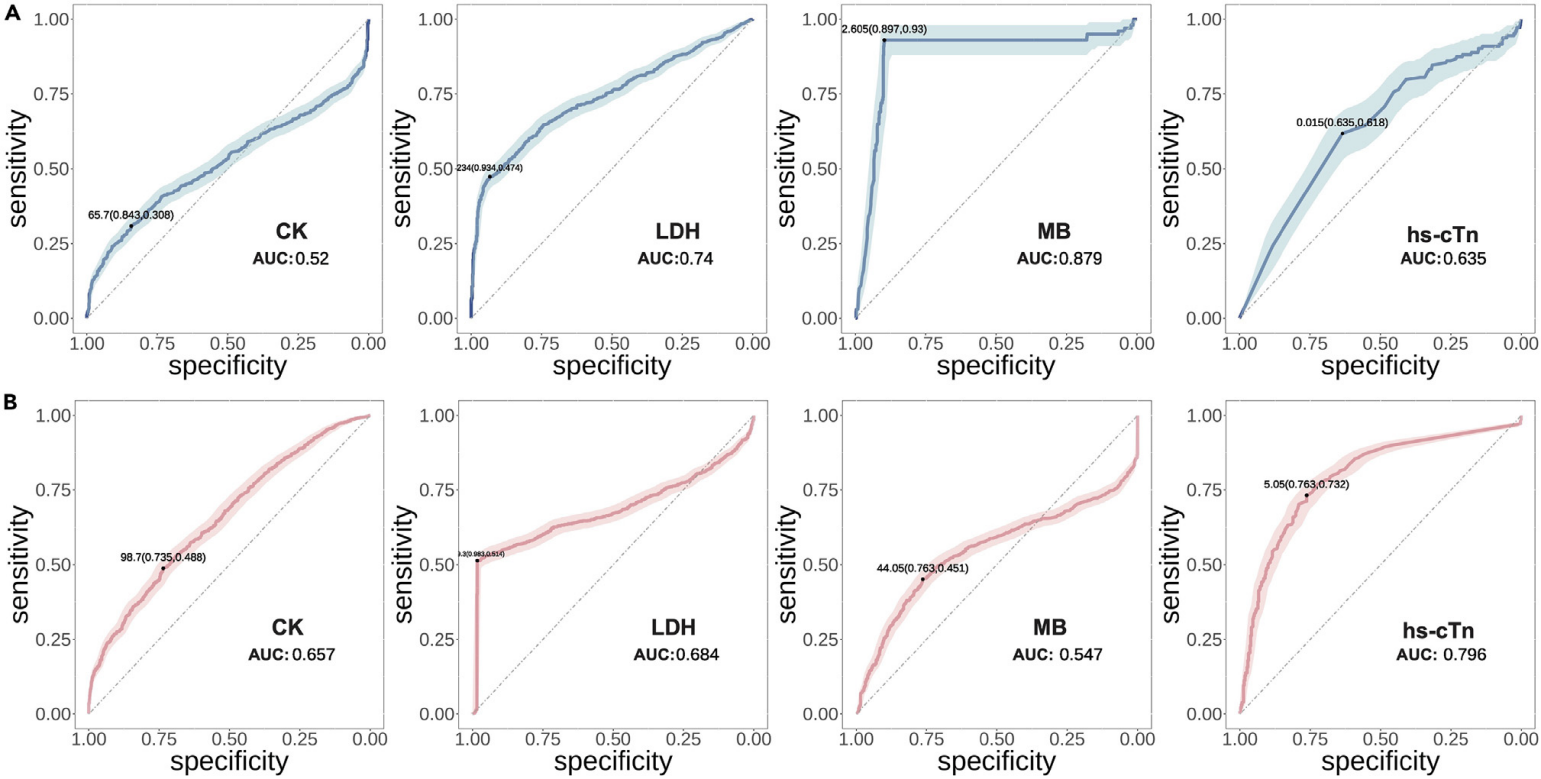
The performance of test results for a single indicator of the predictive model (A) Prediction model results of myocardial marker indicators in healthy individuals and patients with myocarditis by naive Bayes classifie. (B) Prediction model results of myocardial markers in patients with COVID-19 and myocarditis by naive bayes classifier. (Light color is 95% confidence intervals, 25%–75% percentiles, median).

Our data analysis highlighted marked differences in myocarditis relative to healthy individuals and patients with COVID-19. Accordingly, we are trained in a predictive model that employs relevant markers to predict the risk of myocarditis in patients with greater precision. The performance of single index prediction was tested and optimized using a naive Bayes model. We constructed and trained a model using a random forest approach for compound multi-index prediction. Predictive models for myocarditis have been receiving significant attention in contemporary research. These models aim to identify high-risk individuals using a variety of parameters such as clinical symptoms, laboratory test results, and blood analysis. The efficacy of a myocarditis predictive model is appraised based on its sensitivity, specificity, positive predictive value, negative predictive value, and area under the curve (AUC) of the receiver operating characteristic (ROC) curve. The results shown in Figure 6A reflect the performance of myocardial markers in healthy individuals versus those with myocarditis, light color is 95% confidence intervals, 25%–75% percentiles of model predictive performance. Both MB and LDH display commendable performance, with MB having an AUC of 0.887, indicative of high specificity and sensitivity. We concurrently examined the predictive performance of myocardial markers in patients with COVID-19 and myocarditis. As depicted in Figure 6B, the traditional marker, hs-cTn, demonstrates relatively high predictive performance, with an AUC of 0.796, a sensitivity of 0.76, and a specificity of 0.73. Additionally, the CK and LDH markers exhibit predictive solid performance.

**Figure 7:**
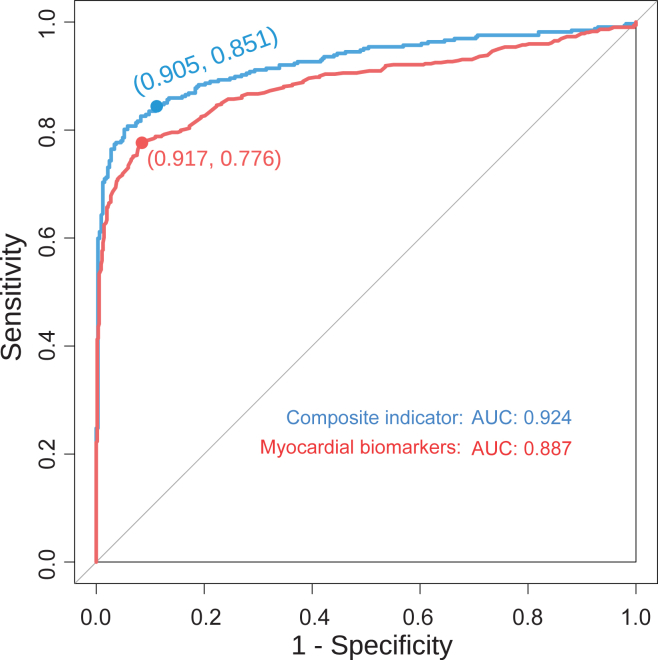
The performance of random forest multi-index hybrid forecasting model The red curve showed the ROC curve of the comprehensive predictive performance of four cardiac markers, and the blue curve showed the ROC curve of ALB, HB, hs-CRP, LYMPHP, NEUTP, PLT, and the multi-indicator predictive performance of four cardiac markers by random forest.

Recognizing the difficulty in accurately predicting a complex disease using a single indicator, we focused particularly on the occurrence of secondary myocarditis following the recovery of patients with COVID-19. To achieve this objective, we analyzed myocardial markers in patients with both COVID-19 and myocarditis. We then employed a random forest machine learning model to perform multi-indicator predictions using a combination of four key myocardial markers. The results, shown by the red line in Figure 7 indicate an AUC of the ROC curve predicted by myocardial markers of 0.887, with a sensitivity of 0.776 and a specificity of 0.917, Youden’s index is 0.693, suggesting good overall performance. For a more comprehensive prediction, we used ALB, HB, hs-CRP, LYMPHP, NEUTP, PLT (Figure S4), and four cardiac markers to train the multi-index predictive model. Under the comprehensive effect of multiple indicators, the performance results of this approach, depicted by the blue line in Figure 7 exceeded those of the cardiac markers alone, with an AUC of 0.924, superior sensitivity of 0.851, and a specificity of 0.905 with Youden’s index is 0.775. It shows that the prediction performance of the model is greatly improved, but also has high sensitivity and specificity.

## Discussion

In our study, we statistically analyzed the correlation and index ratio (AGR, LNR, LPR) of each group of indicators by comparing the data of relevant patients with COVID-19 (Omicron subvariant BA. 5.2, EG.5 in supplemental information) and myocarditis with the healthy population, determined the sample information and index distribution of each group, and conducted single and comprehensive index prediction of myocarditis risk of patients with COVID-19 prognosis by the training Bayesian classification model and the random forest model. It is proven that the prediction models are feasible and have high accuracy. The demographic characteristics of these samples indicate a higher average and median age among patients with COVID-19, along with a male predominance (Figure 2A), this could suggest that older males may be at a higher risk of severe COVID-19. Clark et al.^26^ also came to the same conclusion in their research of the population at increased risk of severe COVID-19 due to underlying health conditions. Conversely, the concentration of patients with myocarditis in the younger age group (Figure 2B) could indicate that this disease may affect younger individuals to a greater extent.^27^ These insights could be valuable for informing public health strategies and clinical management of these conditions.

SARS-CoV-2 may potentially target organ endothelial cells, leading to endothelial dysfunction and resulting in complications such as myocardial injury and thrombosis.^28^^,^^29^ Endothelial dysfunction emerges as a crucial element in understanding COVID-19’s broad impacts. Gustafson et al.^30^ and Fox et al.^31^ also reported on the impact of the SARS-CoV-2 on cardiac abnormalities. The severity of the condition can be assessed by examining blood-related markers such as C-reactive protein, white blood cells and other biomarkers. In their findings, the indicators for patients with myocardial injury were notably elevated, which is in line with our results shown in Figures 4F and S2. Interestingly, a unique feature of patients with COVID-19, as opposed to healthy individuals and patients with myocarditis, is the presence of two distinct indicator distribution communities. Some asymptomatic patients displayed indicators consistent with the healthy population (Figure 3A), while patients with severe COVID-19 symptoms exhibited lower ALB and globulin levels (Figure 3B). The significance of these findings lies in their potential to enhance our understanding of the disease progression and patient stratification in COVID-19.^32^ Among the different sub-variants, patients with EG.5 have fewer instances of low ALB, with a distribution similar to healthy individuals. This suggests that the symptoms in EG.5 patients are milder than those in BA.5.2 patients (Figure S6D). K. Li et al.^33^ found that patients with acute myocarditis had lower levels of ALB and higher levels of GLB compared to healthy controls. Besides, the ALB/GLB ratio was significantly lower in patients with acute myocarditis, suggesting that this ratio may be a valuable biomarker for diagnosing and monitoring myocarditis. Jin et al.^34^ found that the ALB/GLB ratio was significantly lower in patients with COVID-19-associated myocarditis compared to those without myocarditis. The results (Figures 3B and 3C) also clearly showed that the AGR of patients with COVID-19 and myocarditis were significantly lower than that of healthy people, which are consistent with previous research results.^23^^,^^34^^,^^35^ Furthermore, the heatmap and bar chart (Figures 3A–3C) provide a visual and intuitive way to understand the distribution of these indicators, which could be helpful in both clinical and research settings. The clear demarcation in the AGR between healthy individuals and those with COVID-19 or myocarditis could serve as a valuable tool for early detection and intervention.^36^ Overall, these findings underscore the potential utility of the AGR as a diagnostic and prognostic tool in managing COVID-19 and myocarditis. Besides, the dual indicator distribution communities observed among patients with COVID-19 highlight the variable impact of the disease. This could have implications for personalized treatment strategies, where asymptomatic and severely symptomatic patients might require different therapeutic approaches. The findings from Figure 3D have significant consequences for our understanding of the impact of COVID-19 and myocarditis on ALB and TP levels. The marked decrease in ALB levels in patients with these conditions, compared to the healthy population, suggests that these diseases may disrupt the body’s protein balance.^37^ This could potentially lead to a variety of health complications, as ALB plays a crucial role in maintaining fluid balance, transporting substances, and providing the body with the proteins it needs.^38^ The gender differences observed in the healthy population and their absence in patients with myocarditis could suggest that these diseases may affect men and women differently, which was also proved by Fairweather et al.^39^ This could affect how these conditions are diagnosed and treated in different genders. The pronounced difference in protein levels between men and women due to COVID-19 is particularly striking and warrants further investigation.

Currently, LNR and LPR, as new predictive markers, have been used in many studies to predict the risk of myocarditis after recovery from COVID-19.^40^^,^^41^^,^^42^^,^^43^ Bozkurt et al.^44^ studied that within three months after infection, the admission rate of myocarditis in their center is more than ten times that of the vaccination period. Therefore, myocarditis can basically be regarded as the main sequela of COVID-19. The LNR, calculated from standard blood tests, measures systemic inflammation and immune response. It has been identified as an independent risk factor for mortality in hospitalized patients with COVID-19.^40^^,^^41^ In severe COVID-19 cases, patients often have a lower LNR due to an increase in neutrophil count, a response to infection and inflammation, and a decrease in lymphocyte count, potentially a result of viral-induced lymphocyte apoptosis or redistribution.^45^ The scattering analysis of neutrophils and lymphocytes showed a strong linear relationship in the healthy population (Figures 4A–4C). By comparing the two sub-variants of SARS-CoV-2, the LNR distribution of BA5.2 and EG.5 is basically consistent, and the slope value of fitting is also similar (Figure S6E). However, this correlation weakened in patients with COVID-19 and myocarditis, possibly due to disease-related scatter distribution.^40^ The slopes of the fitted linear models for health, COVID-19, and myocarditis were 0.83, 0.82, and 0.74, respectively, indicating a decreasing trend in the LNR with disease severity. It is important to note that the density of hot spots for patients with COVID-19 and myocarditis was concentrated in areas with low lymphocyte ratios, suggesting an increase in disease severity. Our study also looked at individual indicators, such as NEUTP, PLT, and LYMPHP, in men and women. It found statistical significance only in the LYMPHP of patients with COVID-19. The indicators did not show significant differences between patients with COVID-19 and myocarditis, but there was a significant difference when these groups were compared with healthy individuals. The LPR, another ratio calculated from routine blood tests, provides insight into a person’s immune and coagulation status. It has been explored as a predictive marker in various diseases.^46^ Early studies suggest that the LPR could be a valuable tool in predicting the severity of COVID-19, with severe cases often showing a lower LPR due to a decrease in lymphocyte count (lymphopenia) and an increase in platelet count (thrombolysis) in Figures 4G–4I. This imbalance could be indicative of a severe inflammatory response and hypercoagulable state.

Our research has demonstrated that CK levels tend to be elevated in patients with myocarditis (Figure 5A). Increased CK levels indicate myocardial damage, as CK is an enzyme found predominantly in the heart muscle.^47^ However, it is essential to note that elevated CK levels can also be observed in other cardiac conditions.^48^ Despite this limitation, CK remains a valuable biomarker in the overall assessment of myocarditis. Our study has indicated that LDH levels are elevated in myocarditis cases, reflecting tissue damage and inflammation. However, LDH is not specific to myocarditis and can also be elevated in other conditions. Besides, MB is a cardiac-specific isoform of CK and is released into the bloodstream when there is damage to the heart muscle. Research has shown that elevated levels of CK-MB are associated with myocarditis. However, it should be noted that CK-MB can also be elevated in other cardiac conditions and is not exclusive to myocarditis. Our results demonstrated that hs-cTn levels are elevated in myocarditis cases, suggesting ongoing myocardial injury, its high sensitivity enables the early detection of myocarditis and helps assess disease severity.^49^ However, we can clearly observe that EG.5 patients, despite having milder symptoms, have higher levels of hs-cTn (Figure S6F), this suggests that the mutated virus might have reduced pathogenicity in the respiratory system, but enhanced pathogenicity in terms of epidermal barrier disruptions and myocardial damage. Continued research in this area will contribute to refining and optimizing myocarditis diagnosis and treatment strategies. The burgeoning field of machine learning (ML) has ushered in revolutionary capabilities in clinical medicine. Their vast potential in diagnostic and prognostic contexts is gaining traction, particularly in cancer diseases,^50^^,^^51^ and the challenging domain of COVID-19.^52^ However, the actual deployment of AI and ML in clinical decision-making is not without its challenges. The precision and reliability of these tools are contingent upon the comprehensiveness and quality of the data they are trained on. As observed in infectious disease research, many ML models are constructed on data from high-income countries, possibly skewing their efficacy in diverse socioeconomic settings.^50^ Furthermore, while algorithmic performance metrics such as sensitivity and specificity are impressive, the real-world applicability and interpretability of these systems remain paramount concerns. Diving into our research, after comparing the acute myocarditis and COVID-19 groups, it is evident that the ROC of MB and LDH (Figure 6A) differ substantially between the two groups. This variance is further corroborated in the statistical significance analysis showcased in Figure 5A. Notably, the levels of MB and LDH in the COVID-19 and myocarditis groups do not present with statistical significance, leading to the stark divergence in their respective ROC curves.

Our composite multi-index prediction model (Figure 7), which amalgamates a medley of indicators including ALB, HB, hs- CRP, LYMPHP, NEUTP, PLT (as delineated in Figure S4), and a quartet of cardiac markers, elevates the predictive acumen for myocarditis risk in patients. This watershed moment in myocarditis predictive modeling harbors the promise of pinpointing high-risk individuals at an early juncture, thereby catalyzing timely interventions and ameliorating patient outcomes. Moreover, this model holds significant potential in surveilling patients with COVID-19 for emergent secondary myocarditis, equipping clinicians with a tool to preclude further health complications. In essence, our endeavors provide a deeper insight into the nexus between myocarditis and COVID-19, fortifying our arsenal to navigate and neutralize this intricate and capricious condition.

In conclusion, older males may be more susceptible to severe COVID-19, while myocarditis affects younger individuals more. Two distinct communities of indicator distribution were found among patients with COVID-19, with asymptomatic patients exhibiting markers similar to a healthy population. In contrast, those with severe symptoms had lower ALB and GLB levels. These findings inform disease progression understanding and patient stratification. Our research identified the LNR and LPR as potentially useful predictive markers for post-COVID-19 myocarditis risk, which caused the ratio to drop. However, a pronounced increase in myocarditis cases post-COVID-19 infection was noted, underlining its significance as the sequelae of COVID-19. Certain markers (CK, LDH, MB, and hs-cTn) were found to be elevated in patients with myocarditis, indicating myocardial damage. Notably, the composite multi-index prediction model demonstrated significantly improved predictive power for myocarditis risk in patients. This study provides vital insights into myocarditis and its correlation with COVID-19, potentially facilitating earlier identification of high-risk individuals paving the way for timely intervention and improved patient outcomes.

### Limitations of the study

Our study focused on specific COVID-19 subvariants (Omicron subvariant BA. 5.2, EG.5), which may not represent the broader population. Second, this study primarily relied on blood markers, excluding other potential risk factors. Additionally, the sample size, although substantial, may not encompass the full spectrum of COVID-19 and myocarditis cases. External validation is necessary to assess the generalizability of the predictive models. Lastly, the study does not consider evolving COVID-19 variants or long-term effects. These limitations underscore the need for further research for comprehensive insights.

## STAR★Methods

### Key resources table

**Table:** 

REAGENT or RESOURCE	SOURCE	IDENTIFIER
**Critical commercial assays**		

LABOSPECT 006 Automatic Biochemical Analyser	Hitachi, Ltd., Tokyo, Japan	https://www.hitachi.com/
Coulter Ac•T 5diff AL (Autoloader) Hematology Analyser	Beckman Coulter, Ltd, USA	https://www.beckmancoulter.com/
Quantum dots-dry fluorescence immunoassay kit	Kingfocus Co., Ltd., Shenzhen, China	https://www.king-focus.com/en/
The creatine kinase (CK) Activity Assay Kit	Solarbio Life Science Co., Ltd., Beijing, China	http://www.solarbio.net/
Lactate Dehydrogenase Activity Assay Kit	Sigma Aldrich, Ltd, USA	https://www.sigmaaldrich.com/US/en

**Deposited data**		

Covid-19 and Myocarditis patients’ data	First Affiliated Hospital of Guangzhou Medical University, Dazhou Central Hospital	https://data.mendeley.com/datasets/chcnh9g262/1
Code for modeling and analysis	This paper	https://doi.org/10.17632/chcnh9g262.1

**Software and algorithms**		

Python 3.9	Python	python 3.7 version
R-4.2.1	R Core Team	https://mirrors.tuna.tsinghua.edu.cn/CRAN/
Rstudio	R desktop	Rstudio-desktop
sklearn 1.3	python package	https://scikit-learn.org/stable/
naivebayes	R package	https://github.com/majkamichal/naivebayes
Random Forest Classifier	python package	https://scikit-learn.org/stable/modules/generated/sklearn.model_selection.GridSearchCV.html
GridSearchCV	python package	https://scikit-learn.org/stable/modules/generated/sklearn.model_selection.GridSearchCV.html
Adobe Illustrator	Adobe	https://www.adobe.com/de/products/illustrator.html
TBtools	Chen et al.^53^	https://github.com/CJ-Chen/TBtools-II

### Resource availability

#### Lead contact

Further information and requests for resources and reagents should be directed to and will be fulfilled by the lead contact, Baoqing Sun (sunbaoqing@vip.163.com).

#### Materials availability

This study did not generate new unique reagents.

#### Data and code availability

All data reported in this paper will be shared on Mendeley Data: https://data.mendeley.com/datasets/chcnh9g262/1.

All statistical analyses were run in R 4.2.1 using code that implement the algorithms described in the papers that introduced these statistical tests Code available at Mendeley Data: https://doi.org/10.17632/chcnh9g262.1.

Any additional information required to reanalyze the data reported in this paper is available from the lead contact upon request.

### Experimental model and study participant details

The study’s sample comprised 5,230 COVID-19 patients (Omicron subvariant BA. 5.2 and E.G. 5) and 562 myocarditis patients, all of whom had recovered from COVID-19 between 1st December 2022 and 30th March 2023, all the sample were from Chinese. For variance control, we collected data from 730 EG.5 subvariant blood samples between July 15, 2023, and September 15, 2023 in supplemental information (Figure S6). These individuals had giveninformed consent to participate in the study. These patients were from two hospitals: the affiliated hospital of Guangzhou Medical University and Dazhou Central Hospital. The gender and age distribution of the sample is depicted in Figure 2. Venous blood samples were collected from these patients, who had been medically tested and radiologically confirmed to have had COVID-19. Heparin was used as an anticoagulant during the collection process. The samples’ corresponding index data were obtained using an automatic blood biochemical analysis instrument. The myocardial index and other markers were measured using the relevant kit. Any surplus blood was stored at 4 ˝C, centrifuged at 3000 rpm within 30 min of collection, and the supernatant was cleansed and packed before being stored at −80 ˝C for future use. In addition, 4,012 blood samples were randomly collected from healthy individuals who served as controls. In Figure 2B, the age distribution of the healthy population is uniform, and most of the patients with COVID-19 are elderly patients. Still, strangely, most of the patients with myocarditis, a sequela of COVID-19, are young people, and the proportion of people aged 20–30 years is the highest. This sequela has also become a significant threat to the lives of young people.

### Method details

The outcomes evaluated in this study included Serum albumin (ALB), total protein (TP), hemoglobin (HB), blood platelets (PLT), neutrophil proportion (NEUTP), lymphocyte proportion (LYMPHP), and inflammation indicators such as C-reactive protein (CRP), high-sensitive C-reactive protein (hs-CRP), and myoglobin (MB), were meticulously collected from patients’ blood. This data was procured using the LABOSPECT 006 Automatic Biochemical Analyser, a Hitachi, Ltd., Tokyo, Japan product. This sophisticated device can detect a wide range of substances, including ionic lipids, proteins, enzymes, hormones, and other metabolites. Moreover, the granulocyte count of the patients was determined using the Coulter Ac•T 5diG AL (Autoloader) Hematology Analyser, a product of Beckman Coulter, Ltd, USA. Medical System Biotechnology Co., Ltd., Ningbo, China, supplied the test kit for this purpose. Besides, the cardiac biomarkers of high-sensitive cardiac troponin-I (hs-cTn) were measured using a quantum dots-dry fluorescence immunoassay kit, a Kingfocus Co., Ltd., Shenzhen, China product. The creatine kinase (CK) Activity Assay Kit from Solarbio Life Science Co., Ltd., Beijing, China, was employed to measure Creatine Kinase. Lastly, the lactate dehydrogenase (LDH) levels were tested using the Lactate Dehydrogenase Activity Assay Kit, a product of Sigma Aldrich, Ltd, USA.

### Quantification and statistical analysis

Continuous variables were reported as median [interquartile range (IQR)] or mean [standard deviation (SD)] values and were compared across groups using the Wilcoxon rank-sum test or Student’s *t* test. p values were used for description rather than inference. The Hodges-Lehmann estimation was used to determine the 95% CI of the median differences for the non- normally distributed variables. The correlation coefficient measures the relationship between variables, with a positive correlation represented by a coefficient of 1 and a negative correlation represented by a coefficient of −1. Loess regression (local weight regression) for fitting the indicator distribution. The Kruskal–Wallis test is just the rank-sum test extended to more than two samples. In Table 1 the Kruskal Wallis test was used to test patients with COVID-19 and myocarditis, and corresponding p values between the two groups were obtained. Naive Bayes classifiers are a type of simple statistical classifiers that use Bayes’ theorem and assume strong independence between features^54^ and the conditional probability from the model can be decomposed as follows:

(Equation 1) $$P\left(y|x\right)=\frac{P\left(y\right)\cdot P\left(x|y\right)}{P\left(x\right)}$$

Where: P(y|x) is the posterior probability of class (target variable) given predictor (feature), P (y) is the prior probability of a class, P(x|y) is the likelihood which is the probability of predictor given class, P (x) is the "probability of the predictor. In the field of model research, the receiver operating characteristic (ROC) curve is a commonly used tool for evaluating the effectiveness of a model and determining its practical value.^55^^,^^56^ The "pROC" package (version 1.18) can display the ROC curve and calculate the area under the ROC Curve (AUC), which is a critical index in the ROC curve that measures whether positive outcomes are ranked higher than negative outcomes.

#### Prediction model development and validation

Our classification model is grounded in Bayes’ theorem, the Naive Bayes model is particularly effective for large datasets and performs well in complex scenarios due to its simplicity and speed. It’s especially useful when the dimensionality of the inputs is high, which is often the case in medical data. We normalised basic blood indicators to facilitate a more comprehensive understanding of the data. Normalisation is a scaling technique that adjusts the values measured on different scales to a notionally standard scale. This data was followed by “naivebayes” algorithm randomly dividing the data into a training set (70%) and a validation set (30%). The training set is used to build the model, while the validation set is used to test the model’s predictive power. According to Octaviani and Rustam^57^ there is a positive correlation between the prediction accuracy of the Random Forest (RF) model and the number of training sets used. The RF model is a popular machine learning algorithm that operates by constructing multiple decision trees and outputting the class, which is the mode of the categories or mean prediction of the individual trees. We implemented a Python 3.9 RF model using the "sklearn" library version 1.3 to optimise the results. The "GridSearchCV" module was employed to fine-tune the parameters of the RF model. This module exhaustively searches over specified parameter values for an estimator. We selected approximately 200 trees with 15 variables each and a maximum depth of 50. We gathered the results, selected the most efficient model, and evaluated its prediction accuracy using the test set. The number of variables chosen for each tree was also optimised. We employed cross-validation during the parameter adjustment process to prevent overfitting and ensure the model’s stability and practicality. Cross-validation is a resampling procedure used to evaluate machine learning models on a limited data sample. The model’s discrimination performance was evaluated using the Receiver Operating Characteristic (ROC) curve and the associated Area Under the Curve (AUC) value. The ROC curve is a plot that illustrates the diagnostic ability of a binary classifier system as its discrimination threshold is varied. At the same time, the AUC measures the entire two-dimensional area underneath the ROC as a whole curve.

#### Data visualization

This study utilised various tools and techniques for data acquisition, statistical analysis, and visualisation. The primary software used for these tasks were R Core Team version 4.2.1 and Python 3.9, both widely used in the scientific community for their versatility and robustness. A range of graphical visualisations was generated to effectively communicate the findings, with specific software and packages employed for each type of visualisation. We used Adobe Illustrator to optimise color and typesetting, which is crucial for creating clear and aesthetically pleasing visualisations. This software provides a wide range of tools for creating and editing vector graphics. Column charts and box diagrams were created using the "Matplotlib" Python package (version 3.5). Additionally, pair plots were visualised with the assistance of the "seaborn" Python package (version 0.11.2). The heatscatter and statistically significant were generated using the "ggplot2" package (version 3.3.6) and the "LSD" package (version 4.1–0) in R. Tai chi distribution map was made by TBTools software.^53^ In order to compare differences between male and female medical records of various cancers, the "beanplot" package (version 1.3.1) was employed. Lastly, the correlation coefficients between data indices in this study were visualised using the "ggcorrplot" package (version 0.1.3).

#### Ethics

This study was conducted in line with the Declaration of Helsinki. It was approved by the Scientific Research Project Reviews Ethics Committee of the First Affiliated Hospital of Guangzhou Medical University (Document 2021 No. K25) and the ethics committee of Dazhou Central Hospital (protocol code 2023(034)).
