# Supplementary material for: Decoding acute myocarditis in patients with COVID-19: Early detection through machine learning and hematological indices
Source: iScience. 2023 Nov 23;27(2):108524. doi: 10.1016/j.isci.2023.108524 (PMC10831249; doi:10.1016/j.isci.2023.108524)
Supplement: Document S1. Figures S1–S6 and Table S1 [file mmc1.pdf]

**iScience, Volume *27***

## Supplemental information

# Decoding acute myocarditis in patients with COVID-19: Early detection through machine learning and hematological indices

**Haiyang Li, Zhangkai J. Cheng, Xing Fu, Mingtao Liu, Peng Liu, Wenhan Cao, Zhiman Liang, Fei Wang, and Baoqing Sun**

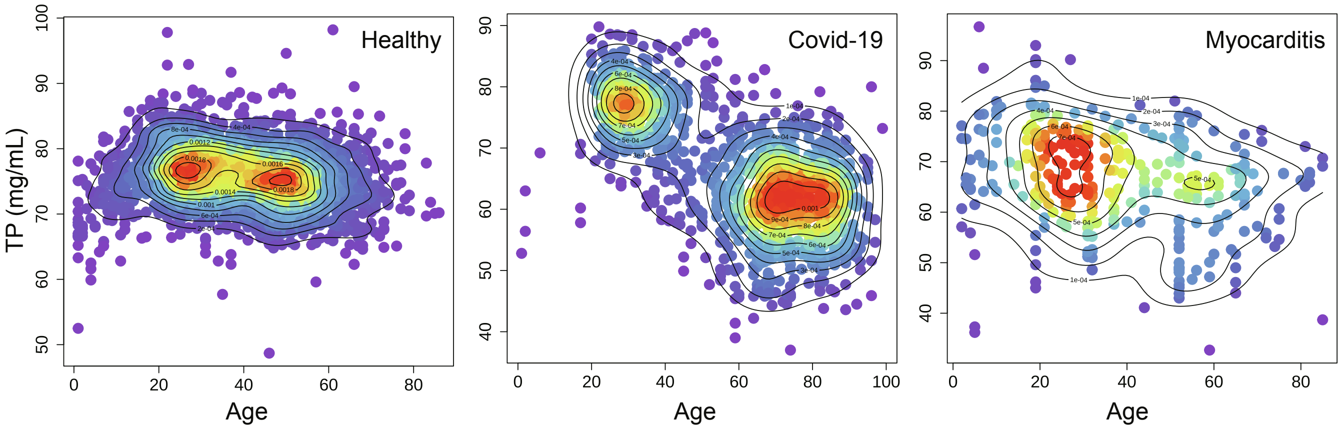


**Supplementary Figure 1  The correlation analysis results of the samples.** The groups are the healthy population, COVID-19 patients and myocarditis patients, respectively.

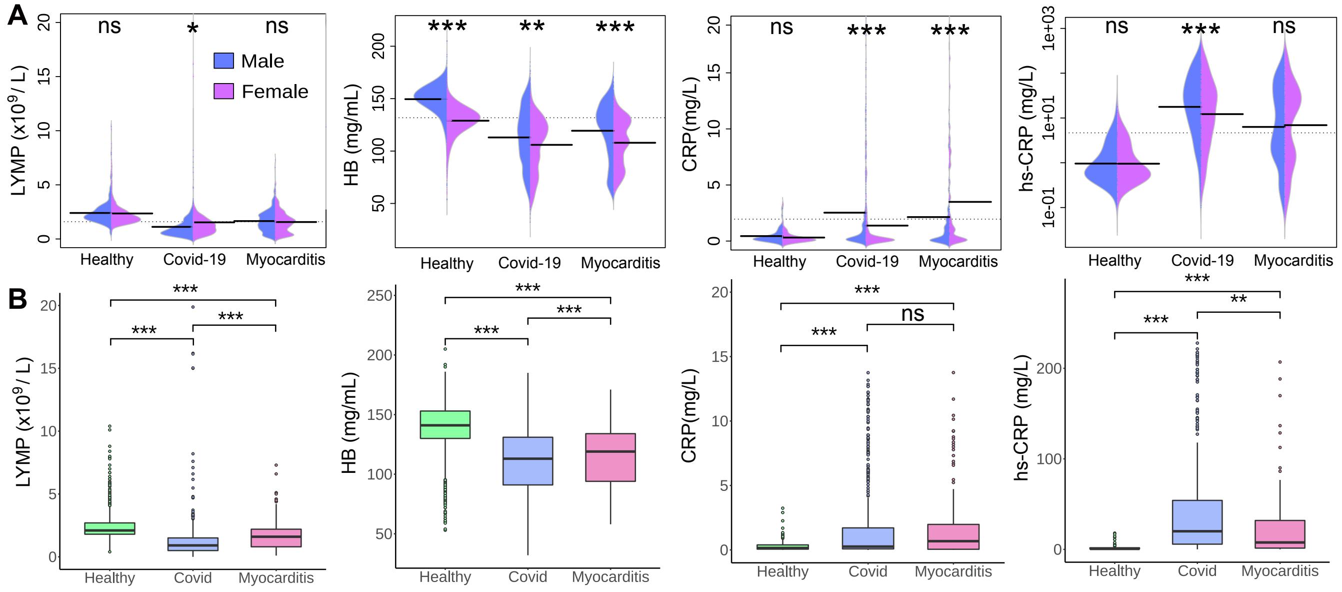


**Supplementary Figure 2  The distribution and group statistical significance analysis results of a single indicator (LYMP, HB, CRP, hs-CRP) in the healthy population, COVID-19. Myocarditis patients groups. A**) Gender statistical significance distribution. **B**) Group statistical significance distribution. Interquartile range, the mean ± SD and t-test, where a statistically significant difference is denoted by *** ($p < 0.001$), **($p < 0.01$), and * ($p < 0.05$), and non-significance ($p > 0.05$) is denoted by "ns".

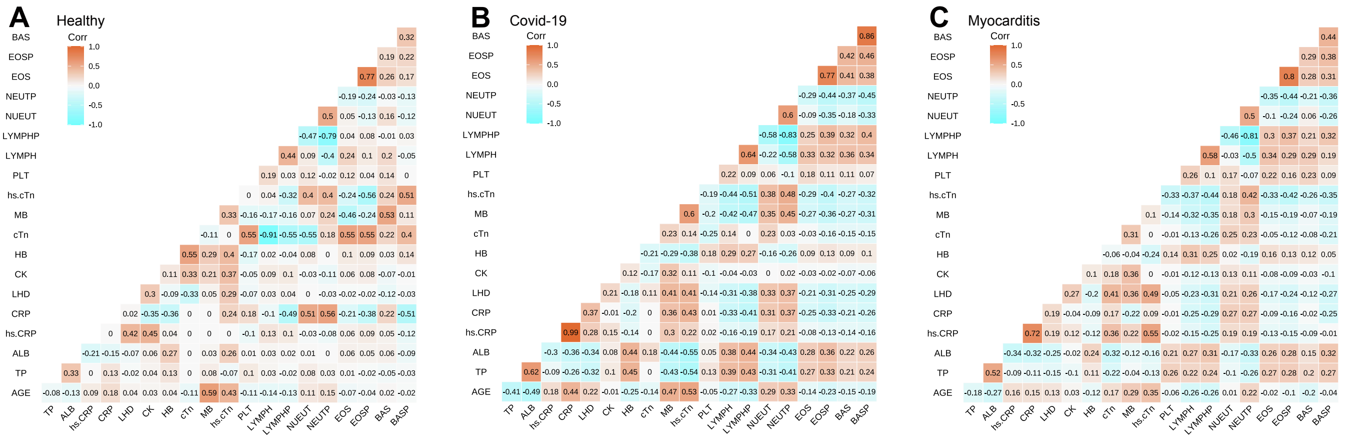


**Supplementary Figure 3  The correlation analysis results of the samples. A**) Healthy population. **B**) COVID-19 patients. **C**) Myocarditis patients.

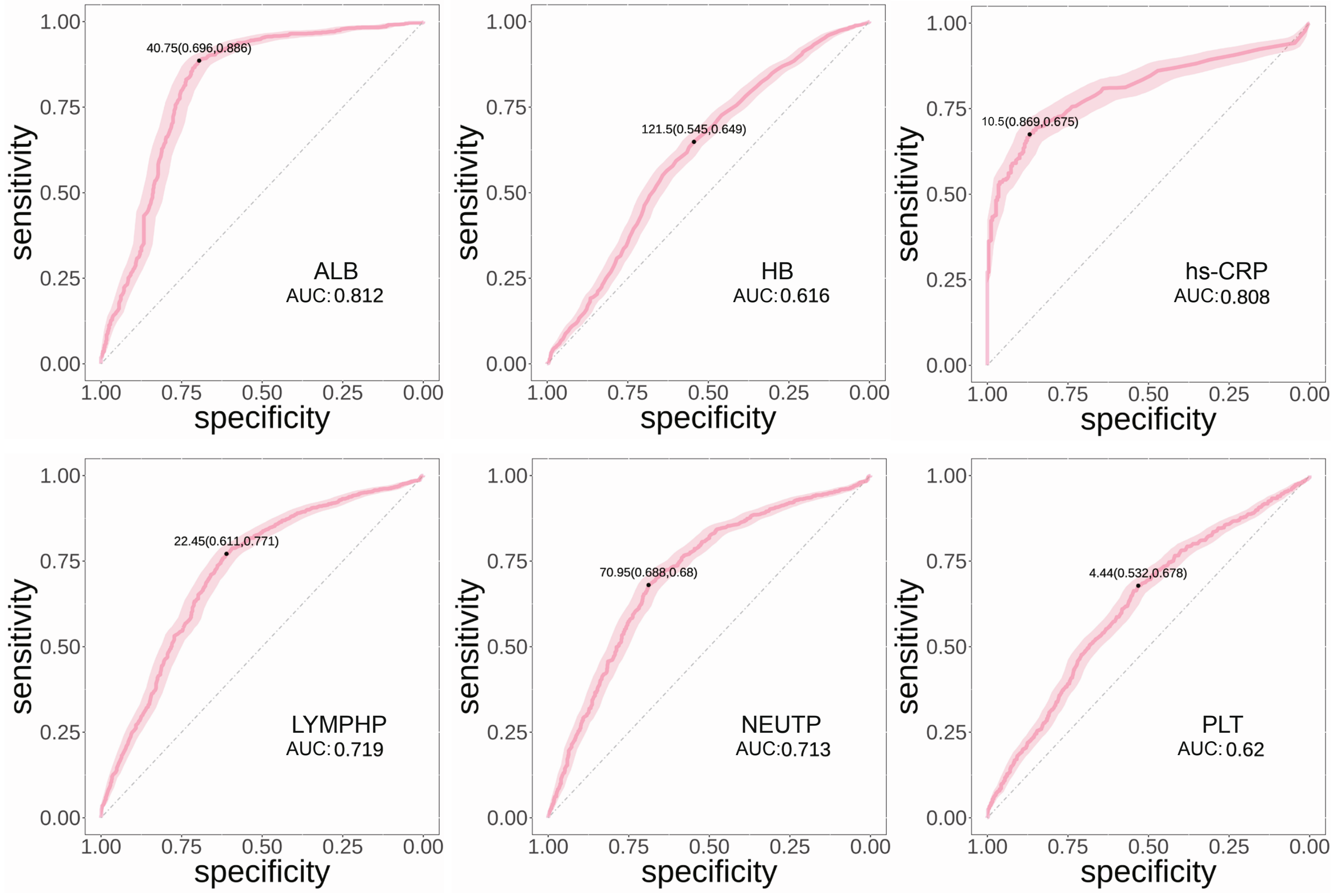


**Supplementary Figure 4  The ROC curve performance of Bayes single index prediction model under different indicators.**

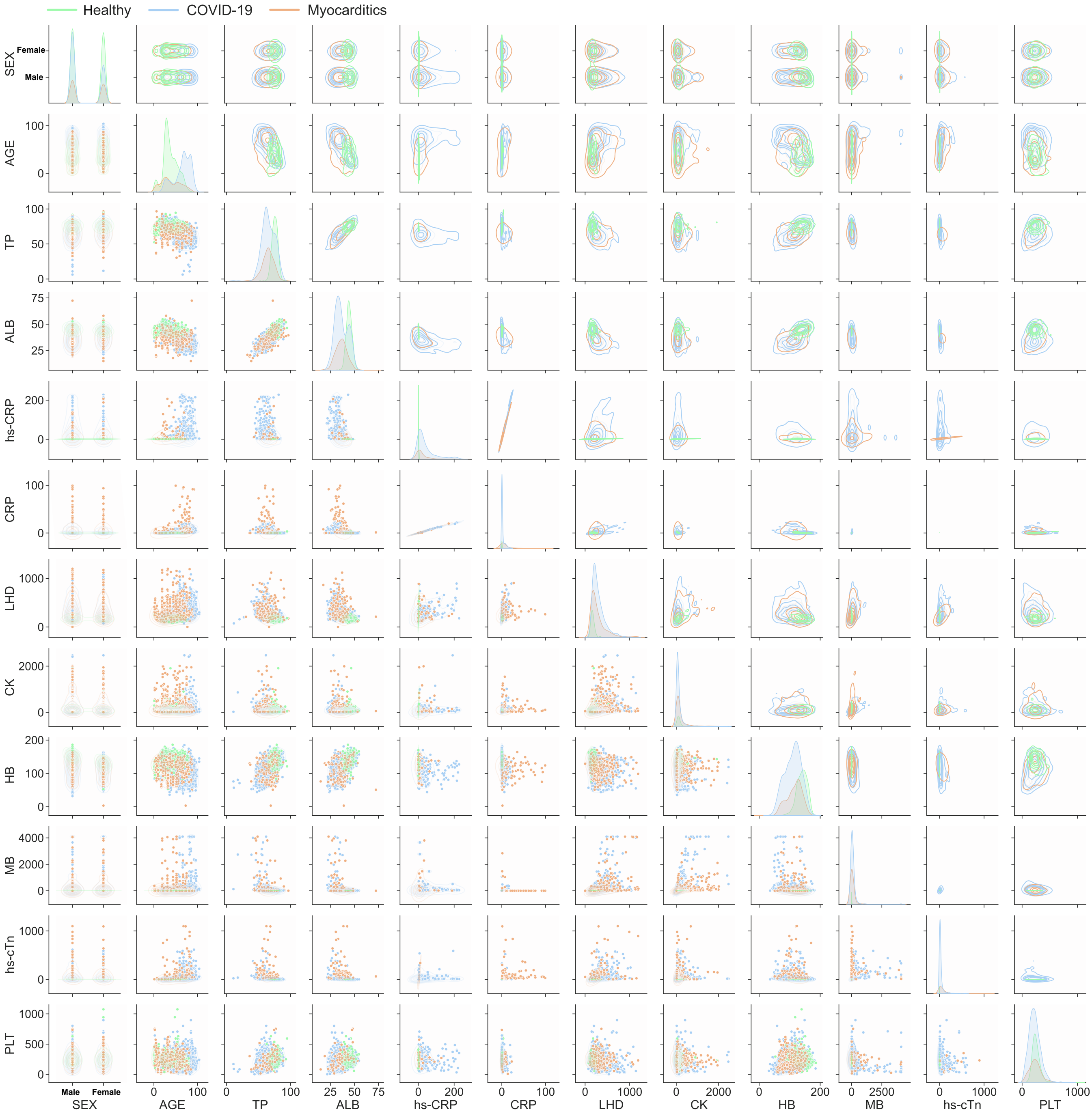


**Supplementary Figure 5 Paired correlation graph between all indicators.** The distribution (lower) and concentrated dispersion (upper) of indicators among each group.

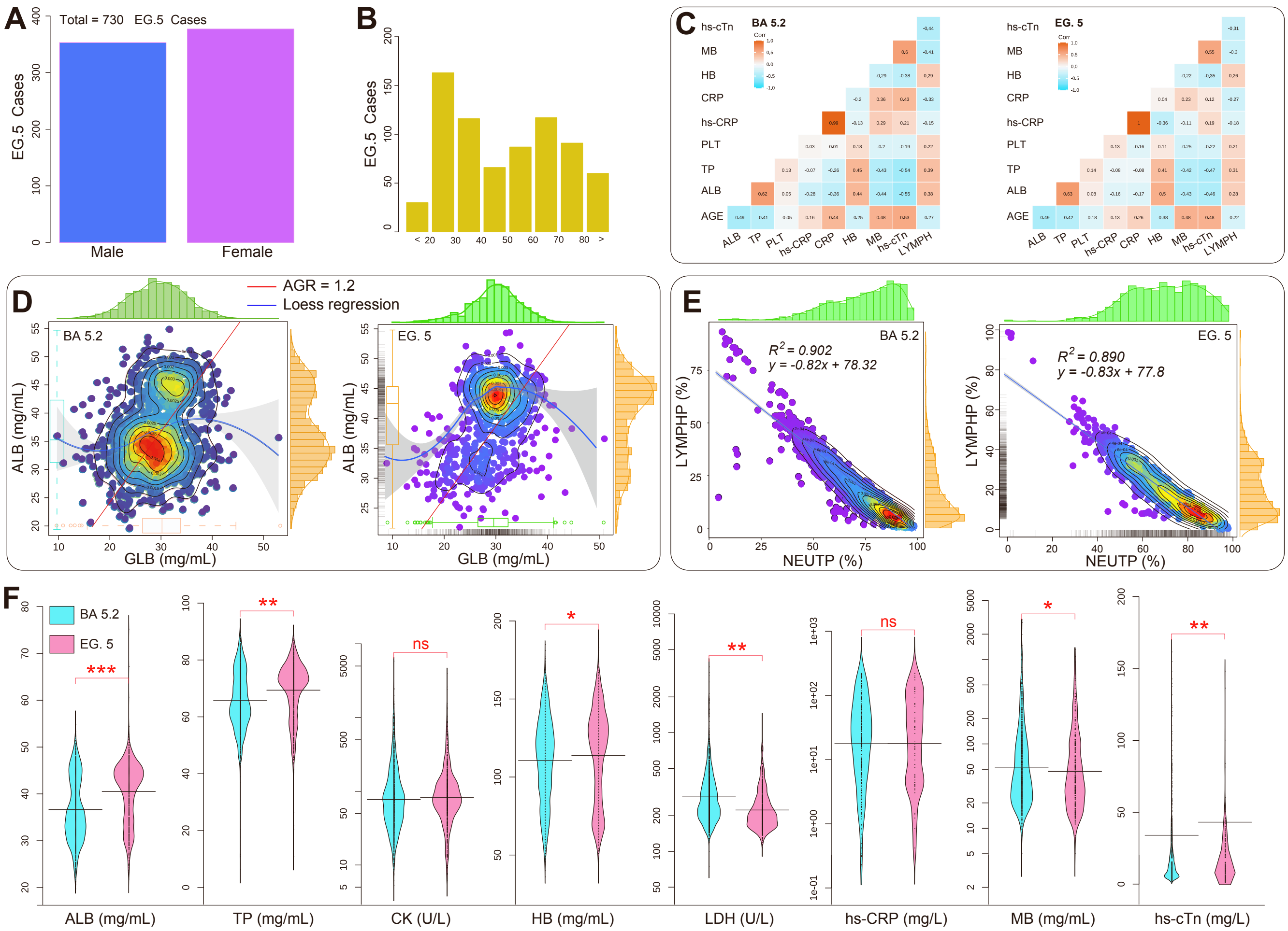


**Supplementary Figure 6 Analysis and comparison of indicators among different COVID-19 variants (BA.5.2 VS EG.5).** **A**) The gender distribution of EG.5 variant participants. **B**) The age distribution of EG.5 variant participants. **C**) The correlation analysis results of BA. 5.2 and EG.5 variant. **D**) The ALB/GLB distribution of BA. 5.2 and EG.5 variant. **E**) The LYMPHP/NEUTP distribution of BA. 5.2 and EG.5 variant.**F**) The significance analysis of differences among indicators of BA.5.2 and EG.5 variant. Interquartile range, the mean ± SD and t-test, where a statistically significant difference is denoted by *** ($p < 0.001$), **($p < 0.01$), and * ($p < 0.05$), and non-significance ($p > 0.05$) is denoted by "ns".

**TABLE S1** The model performance of the single indicator model and the multi-indicator model and the Youden's Index

| Indicator | AUC | Sensitivity | Specificity | Youden's index |
|---|---|---|---|---|
| CK | 0.657 | 0.735 | 0.488 | 0.223 |
| LDH | 0.684 | 0.983 | 0.514 | 0.497 |
| MB | 0.547 | 0.763 | 0.451 | 0.214 |
| hs-cTn | 0.796 | 0.763 | 0.732 | 0.495 |
| ALB | 0.812 | 0.696 | 0.886 | 0.582 |
| HB | 0.616 | 0.545 | 0.649 | 0.194 |
| hs-CRP | 0.808 | 0.869 | 0.675 | 0.544 |
| LYMPHP | 0.719 | 0.611 | 0.711 | 0.322 |
| NEUTP | 0.713 | 0.688 | 0.680 | 0.368 |
| PLT | 0.620 | 0.532 | 0.678 | 0.210 |
| Myocardial biomarkers | 0.887 | 0.917 | 0.776 | 0.693 |
| Composite indicator | 0.924 | 0.905 | 0.851 | 0.756 |
